# A rare intrahepatic subcapsular hematoma (ISH) after laparoscopic cholecystectomy: a case report and literature review

**DOI:** 10.1186/s12893-018-0453-9

**Published:** 2019-01-07

**Authors:** Qiao-fei Liu, Ling-ling Bian, Meng-qing Sun, Rong-hua Zhang, Wei-bin Wang, Yong-ning Li, Jun-chao Guo

**Affiliations:** 1 Department of General Surgery, Peking Union Medical College Hospital, Peking Union Medical College, Chinese Academy of Medical Sciences, Beijing, 100730 China; 2 Department of International Medical Services, Peking Union Medical College Hospital, Peking Union Medical College, Chinese Academy of Medical Sciences, Beijing, 100730 China

**Keywords:** Intrahepatic subcapsular hematoma, Laparoscopic cholecystectomy, Complication

## Abstract

**Background:**

Intrahepatic subcapsular hematoma (ISH) is an extremely rare, life-threatening complication after laparoscopic cholecystectomy (LC). Only few cases have been reported. Herein, we reported a rare giant ISH after LC and summarized all of the reported cases.

**Case presentation:**

A 32-year old woman with recurrent acute cholecystitis for one year, underwent elective LC without intra-operative complications and was discharged 2 days after operation. On the next day after discharge, she developed severe right upper abdominal pain and was sent to our emergency department. The computed tomography scan showed a 10.9 × 12.5 × 6.6 cm ISH in the right liver without free fluid and the hemoglobin dropped to 86 g/l from 127 g/l. Postoperative hemorrhagic shock and a giant ISH after LC were diagnosed. After fluid resuscitation, the hemodynamic was still unstable and the hemoglobin kept dropping. An emergency laparoscopic exploration was performed and the ISH was confirmed, however no active bleeding point was found. A drainage tube was placed under liver for early warning of rupture. Patient was discharged home 10 days after readmission.

**Conclusions:**

Giant ISH is an extremely rare, life-threatening complication after LC. This case showed that the need to consider this rare complication in patients suffering abdominal pain after LC and timely and correct diagnosis and treatment were crucial to saving the lives of the patients.

## Background

Laparoscopic cholecystectomy (LC) substantially reduced the incidence of surgical complications in experienced hands [1]. At present, it is the gold standard treatment for symptomatic cholecystolithiasis patients. The most common complications of LC include iatrogenic bile duct injury, postoperative bleeding, damage to adjacent structures and infections [2]. Intrahepatic subcapsular hematoma (ISH) is an extremely rare complication after LC, which could be life-threatening. Till now, only very few cases have been reported, the experience to diagnose and treat ISH after LC remains largely limited [3, 4]. Herein, we reported a case of giant ISH after LC and reviewed all of the published cases.

## Case presentation

**Fig. 1:**
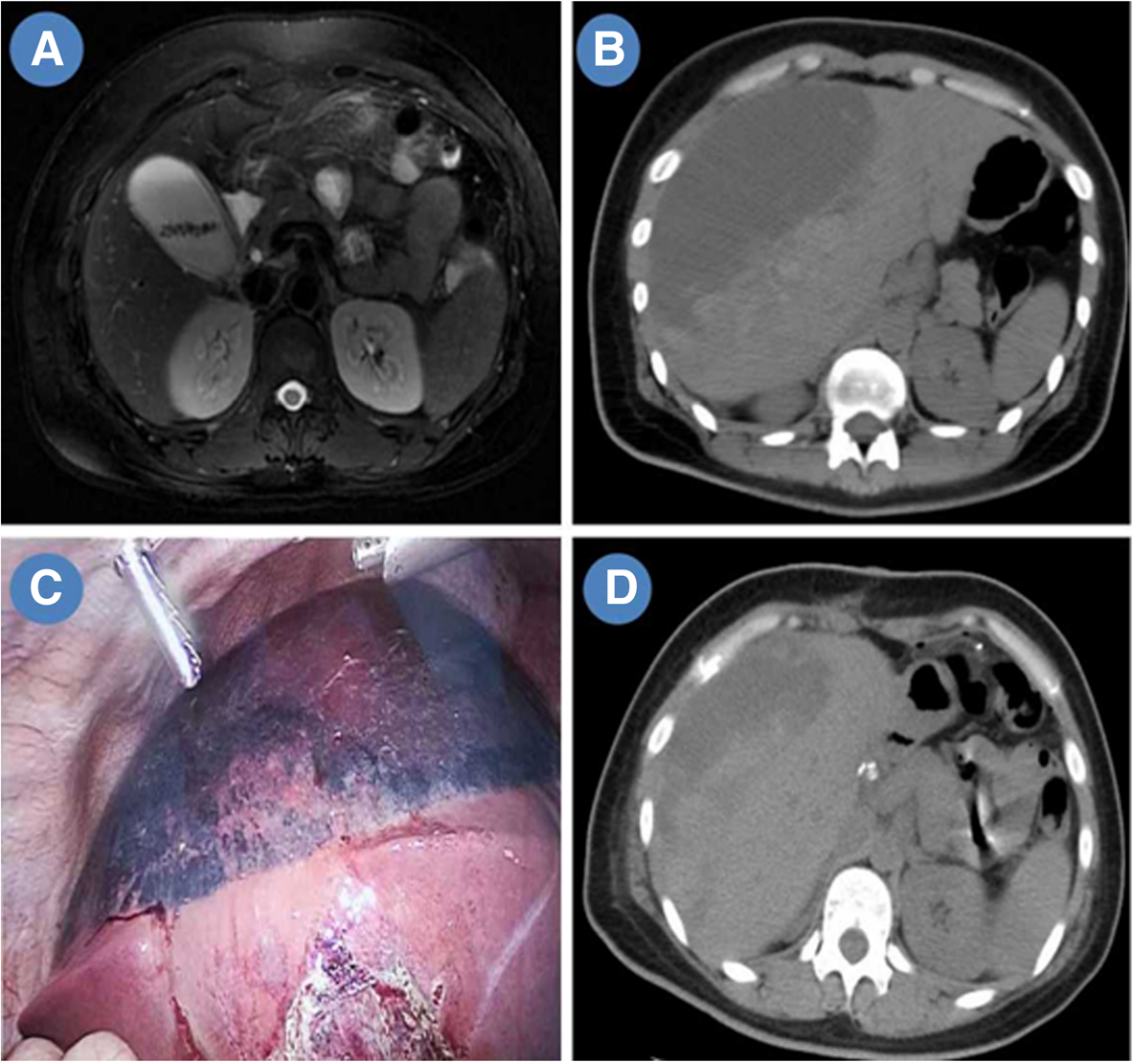
Giant ISH after LC: **a** Multiple gallbladder stones; **b** Giant ISH after LC; **c** Laparoscopic view of a giant ISH; **d** The ISH was absorbed

The patient was a 32-year old woman with a one-year history episodes of cholecystitis treated conservatively. She did not have any other disease history. After an abdominal magnetic resonance imaging that confirmed multiple gallbladder stones (Fig. 1a), an elective LC was performed without intra-operative complications. The recovery was uneventful and the patient was discharged two days after operation. On the second day after discharge, the patient developed severe right upper abdominal pain and she was sent to our emergency department at 8:30 pm. At arrival, her heart rate was 110 bpm and the blood pressure was 80/55 mmHg. The hemoglobin dropped to 86 g/l from 127 g/l. The CT scan showed a 10.9 × 12.5 × 6.6 cm ISH in the right liver without obvious free fluid in abdominal cavity (Fig. 1b). Two hours after fluid resuscitation including 2 U red blood cell, the hemoglobin further declined to 78 g/l and the hemodynamics remained unstable. The abdominal pain was not relieved, after intravenous analgesics. A Doppler ultrasound was performed, two hours later and it found the hematoma had increased in size. Active intrahepatic bleeding was suspected. We called radiologist for consultation, however, the interventional angiography and embolization was not available at mid night. We explained the potential risk of sudden rupture of hematoma during conservative methods which may cause sudden death, to the patient and her relatives. After careful consideration of the continuous decline of hemoglobin, unstable hemodynamics after fluid resuscitation, we explained our surgical plan to the patient and her relative. We planned to perform laparoscopic exploration at first, if the hematoma continued to expand, we would evacuate or drain it, if not, we would put a drainage tube under liver which could serve as an early warning of rupture. The patient requested surgical method to reduce the risk of sudden death. Therefore, an emergency laparoscopic exploration was performed under general anesthesia. The ISH was confirmed (Fig. 1c). Four U red blood cell and 400 ml fresh frozen plasma were transfused. After fluid resuscitation and blood transfusion, her hemodynamic became stable. During the 3-h intra-operative observation, the hematoma did not expand. Therefore, a non-sucking drainage tube was placed under the liver and she was sent to ICU ward. Next morning, she was transferred to the ordinary ward. The upper abdominal pain gradually relieved. Five days after the laparoscopic exploration, another CT scan showed that the hematoma was largely resolved and we removed drain tube (Fig. 1d). She was discharged, 10 days after readmission.

Totally, 13 papers, including 16 cases of ISH after LC were reported from 1994 to 2015 (Table 1). Nearly half of the patients had instability of hemodynamics. All of the cases were female patients. Age of patients ranged from 25 to 78. All hematomas were mainly located in the right lobe of liver, and some of them extended to the left lobe of liver. Only one case was ruptured at diagnosis. Hepatic capsule laceration was found in two cases, one of whom also took NSAIDS (non-steroids anti-inflammatory drugs) to control the pain after operation. Totally, 58.8% of patients took NSAIDS to control the post-operative pain, and most of them used Ketorolac, however, 35.3% of the patients still did not have definitive risk factors. The time interval to diagnose ISH after LC ranged from seven hours to six weeks. They were diagnosed most commonly (35.5%) within one day after LC. All patients had abdominal pain and 47.1% of the cases developed hypovolaemic shock.

**Table 1:** Literature review of ISH after LC

No.	Year of Publication	Gender	Age	Causes	Location of hematoma	Time interval after LC	Symptoms	Treatments	Outcomes
1	2015 [12]	Female	25	No definitive cause	Right lobes of liver	One day after LC	hypovolaemic shock	Laparotomy, hematoma evacuation	13 days after laparotomy, discharged
2	2015 [8]	Female	60	No definitive cause	Right lobes of liver	Six days after LC	Right upper abdominal pain, nausea, fever	Percutaneous drainage under ultrasound guidance	18 days after readmission, discharged
3	2012 [3]	Female	77	Coumarin (anticoagulation drug)	Right lobes of liver	Six weeks after LC	Right upper abdominal pain	Selective embolization of the bleeding vessel, drainage under ultrasound guidance	Recovered uneventfully
4	2011 [13]	Female	25	Capsule laceration	Right lobes of liver extended to left side	Two days after LC	Right upper abdominal pain, drop of HGB	Laparoscopic exploration	Right pleural effusion was treated with a pigtail pleural catheter; 5 days after laparoscopic exploration, discharged.
5	2010 [9]	Female	28	Capsule laceration, NSAIDS	Raptured right liver hematoma	One day after LC	Right upper abdominal pain, hypotension, tachycardia, and severe anemia	Laparotomy, and hematoma evacuation, drainage	Discharged without further complaints
6	2010 [4]	Female	60	Ketorolac (NSAIDS), ultiple myeloma	Right lobes of liver	Six days after LC	Right upper abdominal pain, hemodynamic instability	Laparotomy, hematoma drainage and packing	31 days after laparotmy, discharged
7	2010 [4]	Female	29	Ketorolac (NSAIDS)	Right lobes of liver	One day after LC	Perspiration and hypotension	Laparotomy, hematoma drainage and packing	30 days after laparotmy, discharged
8	2009 [10]	Female	Not available	No definitive cause	Right lobes of liver	Not available	abdominal pain	Conservative treatments	Uneventful recovery
9	2008 [14]	Female	Not available	Ketorolac (NSAIDS)	Raptured right liver hematoma	Not available	abdominal pain, hemodynamic instability	Laparotomy, hematoma evacuation and drainage	Uneventful recovery
10	2005 [11]	Female	61	NSAIDS	Right lobes of liver	Three days after LC	Fever, upper abdominal pain,	Percutaneous drainage under CT guidance	Uneventful recovery
11	2005 [11]	Female	66	NSAIDS	Right lobes of liver	Not mentioned	Fever, upper abdominal pain, nausea	Conservative treatments	Uneventful recovery
12	2004 [15]	Female	64	No definitive cause	Right lobes of liver	Ten days after LC	upper abdominal discomfort, nausea, and pyrexia	Percutaneous drainage under ultrasound guidance	14 days later, discharged
13	1998 [16]	Female	48	Ketorolac (NSAIDS)	Right lobes of liver	One day after LC	upper abdominal discomfort, nausea	Percutaneous drainage under ultrasound guidance, twice	14 days later, discharged
14	1998 [16]	Female	28	Ketorolac (NSAIDS)	Right lobes of liver	Seven hours after LC	Right upper abdominal pain, hypotension, tachycardia, and severe anemia	Laparotomy, and hematoma evacuation, drainage	8 days later, discharged, 3 weeks later, the drainage tube was removed
15	1998 [17]	Female	78	No definitive cause	Right lobes of liver	Not available	Right upper abdominal pain	Conservative treatments	Unenventful recovery
16	1994 [18]	Female	35	Ketorolac (NSAIDS)	Right lobes of liver	Nine hours after LC	Tachycardia and hypotension	Laparotomy	1 week after laparotomy, discharged

Treatment strategies included: conservative treatment (antibiotics, blood transfusion, strict bed-reset), percutaneous drainage under CT or B ultrasound guidance, selective embolization of the bleeding vessel, laparoscopic exploration and laparotomy. Eighteen percent of patients had stable condition without fever and underwent conservative treatments. The only case of angioembolization was complicate by infection and required percutaneous drainage. For the patients with stable condition, fever and serious compression of inferior venal cava (IVC) always were indications for percutaneous drainage under CT or B ultrasound guidance. In these 17 cases, 29.4% of the patients underwent percutaneous drainage. For the patients with hemodynamic instability, emergent reoperation was adopted. Totally, nine cases underwent reoperation, including two case of laparoscopic operation and seven cases of laparotomy. For our case, we only performed laparoscopic exploration and did not perform evacuation or drainage of the hematoma, since the hemodynamic became stable after plenty fluid resuscitation and the hematoma did not expand, during the 3 h of intra-operative observation. For another case, laparoscopic exploration found small capsule laceration, and hemostasis was performed. In the seven cases of laparotomy, six patients underwent evacuation and drainage of hematoma, only one case underwent only laparotomy without evacuation or drainage.

All patients survived. Most of patients stayed one to two weeks after readmission, however, the longest hospital stay was up to 31 days after reoperation.

## Discussion and conclusion

LC is a widely performed procedure for symptomatic gallbladder stone. Although the overall morbidity is less than 7%, it carries certain risks, the most common complications include infections, bile leak and postoperative bleeding [5]. Postoperative bleeding after LC is observed in less than 1% of cases. The most common sites of postoperative bleeding include: gallbladder bed, sites of trocar, cystic artery, falciform ligament and bleeding from the ruptured liver capsule [6]. The ISH is a rare, life-threatening complication after LC. No conclusive cause for ISH has been found. Iatrogenic liver trauma, NSAIDS, anticoagulants, hepatic haemangioma, anatomical variations of the hepatic vascular system have all been named as possible contributing factors [4, 7, 8]. In this case, the patient took Parecoxib to control the pain. The literature review showed that 58.8% of the patients took NSAIDS to control postoperative pain, and most of them took Ketorolac. The preoperative coagulation function of this patient was normal and as well, she did not suffer no steatosis or cirrhosis which may be risk factors for ISH after minor trauma.

When the ISH is small, it is usually asymptomatic, therefore, it is very difficult to detect it at an early stage. However, when it keeps developing, it could be life-threatening, leading to hypovolaemic shock and even death. The treatment for ISH should be adopted according to the condition of the patients. When the ISH is small and the condition of patient is stable, conservative treatment is always preferred. Some other more aggressive treatments, including percutaneous B ultrasound -guided drainage, relaparotomy or relaparoscopy, evacuation or drainage of the hematoma, should be considered when the ISH is too big or the patient is hemodynamically unstable [9–11]. In all of these 17 cases, only one case was ruptured, however, 14 cases were operated, therefore, rupture may not the unique indication for surgical treatment. The most important key point for ISH is the risk of sudden death due to massive bleeding after rupture of hematoma. When the doctors explain the possibility of this lethal outcome to the patients, they always request to reduce this possibility as much as possible. This patient suffered hemodynamical instability and continuous drop of hemoglobin even if after fluid resuscitation and the hematoma became larger after her admission within 2 h. The risk of rupture did exist for this case. When we explained the risk of rupture to the patient, she rejected the conservative treatment and requested surgical methods to reduce the risk of sudden death after potential massive bleeding. Laparoscopic exploration was minimally invasive and a drainage tube under the live would show the bleeding immediately if it occurred. Therefore, we chose the emergent laparoscopic exploration. After a 3-h intraoperative observation, the ISH did not expand and her condition was stable, in the meanwhile, in consideration to the invasive trauma of debridement and uncertain outcome of drainage of ISH, we only chose to place the drain tubes as early warning for rupture. Later, it gradually resolved. Frankly speaking, if we chose conservative treatments, including further liquid resuscitation and blood transfusion, the outcome would be the same, however, no one could guarantee this satisfactory result at that time, especially when the patients did not accept the possibility of sudden death after rupture during conservative treatment. For this giant ISH, in case of rupture in absence of drainage tube for early warning, it could be difficult to detect the bleeding immediately which may cause disastrous result.

Despite LC has been widely performed and the complications of LC is relatively low in the experienced hands, some serious complications can still occur. Giant ISH is an extremely rare, life-threatening complication after LC. The experience of how to treat this kind giant ISH remains largely limited. Herein, we presented a rare case and summarized all of the previously published cases which could substantially increase our experience to treat this rare condition. The most important key point for this giant ISH is to avoid sudden death after the rupture of hematoma, luckily, according to this literature review, only one case was ruptured and no mortality was reported.

## Abbreviations

CT: Computed tomography
ISH: Intrahepatic subcapsular hematoma
LC: Laparoscopic cholecystectomy
NSAIDS: Non-steroid anti-inflammatory drugs
